# Analysis of eight types of RNA modification regulators and their correlation with the prognosis in hepatocellular carcinoma

**DOI:** 10.3389/fgene.2023.1127301

**Published:** 2023-03-16

**Authors:** Wan Qin, Chen Jin, Jun Zou

**Affiliations:** ^1^ Department of Oncology, Tongji Hospital, Tongji Medical College, Huazhong University of Science and Technology, Wuhan, China; ^2^ Department of Epidemiology, Center for Global Health, School of Public Health, Nanjing Medical University, Nanjing, China; ^3^ Department of Medical Oncology, National Cancer Center, National Clinical Research Center for Cancer, Cancer Hospital, Chinese Academy of Medical Sciences and Peking Union Medical College, Beijing, China

**Keywords:** RNA modification, hepatocellular carcinoma, prognosis, nomogram, therapeutic responses

## Abstract

RNA modification plays important role in the occurrence and development of hepatocellular carcinoma. The best characterized RNA modification is m6A, while other kinds of RNA modifications have not been fully investigated in hepatocellular carcinoma (HCC). In the current study, we investigated the roles of one hundred RNA modification regulators belonging to eight different types of cancer-related RNA modifications in HCC. Expression analysis revealed that nearly 90% RNA regulators exhibited significantly higher expression in tumors than normal tissues. By consensus clustering, we identified two clusters with distinct biological characteristics, immune microenvironment, and prognostic pattern. An RNA modification score (RMScore) was constructed and stratified patients into high- and low-risk group, which showed significantly different prognosis. Moreover, a nomogram including clinicopathologic features and the RMScore could well predict the survival in HCC patients. This study indicated the important role of eight types of RNA modification in HCC and develop a RMScore, which will be a new method to forecast the prognosis of HCC patients.

## Introduction

Hepatocellular carcinoma (HCC) is one of the leading cause of cancer-related deaths worldwide, with few effective therapeutic options (Sung et al., 2021). Most patients have already progressed to the middle and late stages when they are diagnosed, leading to a poor prognosis of HCC patients (Sia et al., 2017). The complicated etiologies and complex pathogenesis lead to the high heterogeneity of HCC, making the efficiency of treatment very low (Park et al., 2010; Seehawer et al., 2018). Identifying novel treatment and prognostic targets for HCC is of urgent need.

RNA modification is a critical posttranscriptional regulators of gene expression program (Nombela et al., 2021). With the rapid development of molecular and sequencing technologies, RNA modification has become a hot research topic in recent years. Emerging reports confirm that dysregulation of RNA modification gives rise to a variety of human diseases, particularly hepatocellular carcinoma (Xu et al., 2021). Various types of cancer-related RNA modifications can be found in eukaryotes, including N6-methyladenosine (m^6^A), N1-methyladenosine (m^1^A), and 5-methylcytosine (m^5^C), 2′-O-methylation (Nm), m^7^G N7-methylguanosine (m^7^G), pseudouridylation (Ψ), adenosine-to-inosine (A-to-I), 5-methoxycarbonylmethyl-2-thiouridine (mcm^5^s^2^U) (Boccaletto et al., 2018; Frye et al., 2018). Among them, m^6^A is the most investigated and best characterized one (Shi et al., 2019; Wei and He, 2021). For example, KIAA1429 was demonstrated to promote HCC invasion and migration by altering the methylation of m6A in ID2 and GATA3 mRNA (Cheng et al., 2019; Lan et al., 2019). ALKBH5 suppresses proliferation and invasion of HCC *via* m6A-guided epigenetic inhibition of LYPD1 (Chen et al., 2020). METTL3 promotes HCC progression *via* post-transcriptional silencing of SOCS2 (Chen et al., 2018). Other methylations are also reported to be related to HCC. For example, aberrant NSUN2-mediated m^5^C modification of H19 lncRNA is proved to be associated with poor differentiation of HCC (Sun et al., 2020). TRMT6/TRMT61A-mediated m1A methylation is required for self-renewal of liver cancer stem cells and tumorigenesis (Wang et al., 2021a). However, these studies mostly focused on the functions of only one RNA modification regulator. The crosstalk between different regulators remains unknown. Recently, the interactions between different regulators within one type of RNA modification have been investigated. For example, Fang et al. reported a two m^6^A gene-based signature (HNRNPA2B1 and RBM15) could predict the prognosis of HBV-related HCC (Fang and Chen, 2020). A risk signature consisted of four m^1^A regulators (TRMT6, TRMT61A, TRMT10C, and YTHDF1) was remarkably associated with HCC patient prognosis (Shi et al., 2020). A risk signature comprised of six m^6^A-related genes was reported to be able to predict the prognosis of HCC(19). However, these studies ignored the relationship and crosstalk between different types of RNA regulators. It is very necessary to systematically characterize the relationship and crosstalk of other kinds of RNA modification regulators in HCC.

In the current research, we profiled one hundred RNA regulators from eight types of RNA modifications in HCC for the first time. We clustered HCC patients into two RNA modification-related subtypes with different prognosis and cancer hallmarks. An RNA modification score was constructed to serve as an independent prognosis predictor for HCC patients. These results would provide insight into the prognosis prediction and therapeutic management of HCC in the future.

## Materials and methods

### Data collection

The gene mutation, expression and clinical information of HCC patients was downloaded from the TCGA and ICGC database.

There were huge number of RNA regulators identified in eukaryotes. However, we only choose RNA regulators which were proved to play critical roles in HCC and other cancers (Delaunay and Frye, 2019; Barbieri and Kouzarides, 2020; Han et al., 2021). As a result, a total of one hundred RNA regulators which belong to eight types of RNA modifications (m^6^A, m^1^A, m^5^C, Nm, m^7^G, Ψ, A-to-I, and mcm^5^s^2^U) were included in our study. They were listed in Supplementary Table S1.

### Expression and mutation analysis of RNA modification regulators

Unpaired *t*-test were used to compare the expression of 100 RNA regulators between tumor and adjacent normal tissues. The “Maftools” R package was used to analyze the mutation and interaction of 100 RNA modification genes. The protein-protein interaction between 100 RNA modification regulators were analyzed by the STRING database and visualized by using Cytoscape software (Szklarczyk et al., 2019).

### Identification of RNA modification regulator-related subgroups

To figure out the relationship between the expression of 100 RNA modification regulator and HCC subtypes, consensus clustering analysis was conducted in TCGA-LIHC patients by using the “ConsensusClusterPlus” R package.

### Pathway enrichment analysis

To reveal the biological function enriched in different group of patients, the Gene Set Variation Analysis (GSVA) was performed by utilizing the R package “GSVA”. The annotated gene set “h.all.v7.4.symbols.gmt” was used for GSVA analysis. The enrichment scores of immune cells in sample from different groups were determined with single-sample gene set enrichment analysis (ssGSEA).

### Construction and validation of the RNA modification score (RMScore)

To construct the RMScore, we first identified 1,427 differentially expressed genes (DEGs) in cluster 1 and cluster 2 groups. We divided TCGA-LIHC into training set and validation set. In the training set, univariate cox regression analysis revealed that 266 DEGs were identified to be significantly related to the prognosis of HCC patients (*p* < 0.001). Then LASSO regression analysis was conducted to select survival-associated DEGs. Multivariate cox regression analysis was conducted to construct the final prognostic model. The RMScore was calculated by the following equation: RMScore = Exp1* Coe 1 + Exp2* Coe 2 + … Exp N* Coe N.

The validation dataset of TCGA-LIHC, whole TCGA-LIHC dataset, and ICGC dataset were utilized as external validation dataset. The RMScore of each sample was calculated by the formula presented above. Patients were divided into low RMScore and high RMScore groups by the median value of the RMScore. The “Survival” R package was conducted to analysis the survival of two groups. The “SurvivalROC” R package was used to plot the ROC curves.

### Chemotherapy response prediction

To predict the sensitivity to chemotherapeutic drugs, we use the R package “pRRophetic” to estimate the half-maximal inhibitory concentration (IC50) in individual patients. The R package “ggplot2” was utilized to generate the plots.

### Construction of a nomogram

The stage and RMScore, both of which were identified to be an independent prognostic value by multivariate Cox proportional hazards analysis, were included to construct the nomogram. The calibration curve was plotted to observe the prediction probabilities of the nomogram against the observed rates. The prognostic ability of the nomogram was evaluated by receiver operating (ROC) curves and decision curve analysis (DCA) curves.

## Results

### Dysregulation and mutation of eight types of RNA regulators in HCC

We choose a total of one hundred RNA modification regulators which were reported to play roles in at least one kind of cancer to perform following analysis (Supplementary Table S1).

**FIGURE 1:**
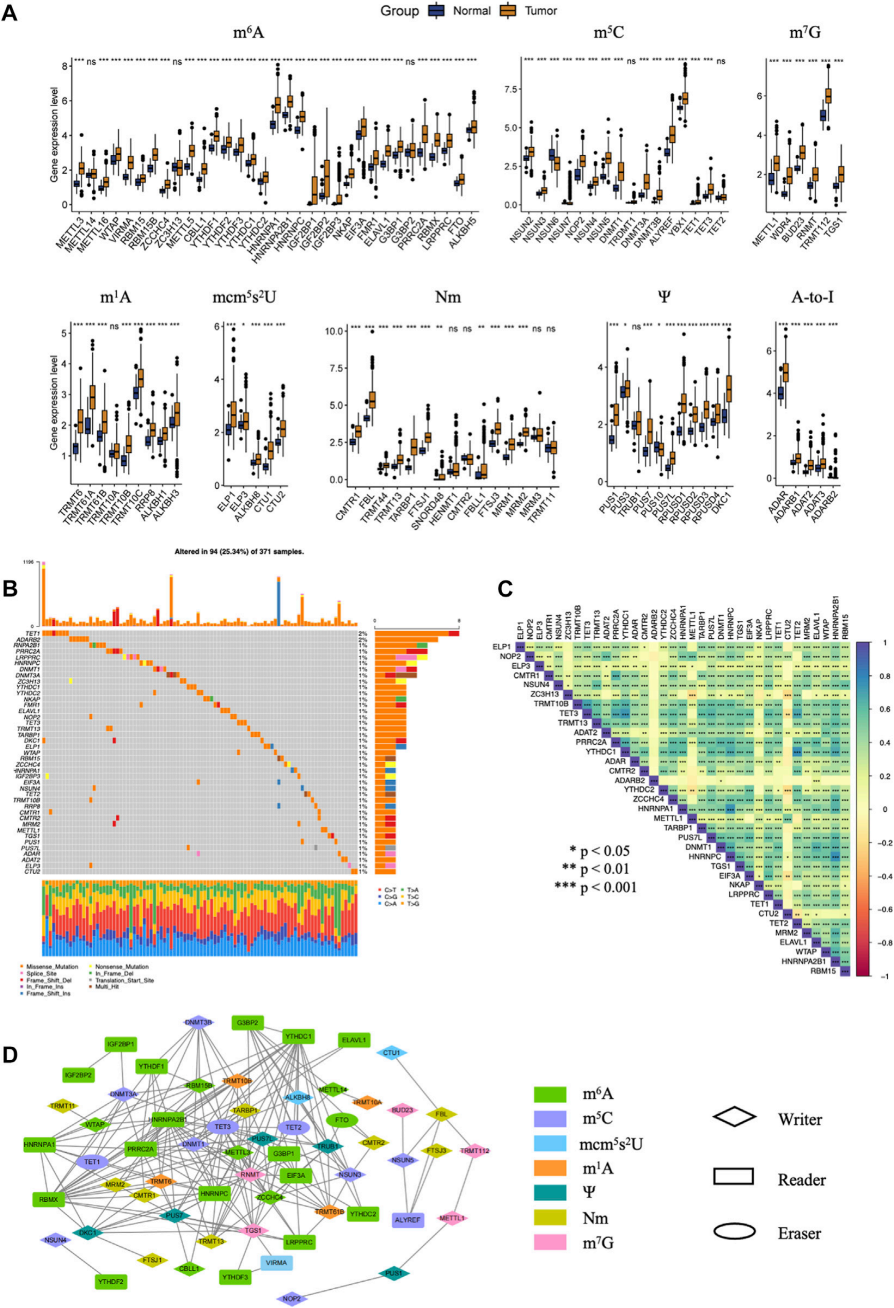
The dysregulation and mutation landscape of 100 RNA modification regulators included in our study in HCC. **(A)** The gene expression level of 100 RNA modification regulators in normal and tumor tissues in TCGA-LIHC. **(B)** Genomic mutational landscape of RNA modification regulators with mutation rates ≥1% in the TCGA-LIHC cohort. **(C)** Correlation of the mutation between genes with mutation rates ≥1%. **(D)** The protein-protein interactions between one hundred RNA regulators analyzed by the STRING database. (*p*-value < 0.05 and correlation coefficient ≥0.6). (ns, not significant; *, *p* < 0.05: **, *p* < 0.01; ***, *p* < 0.001).

In order to characterize the expression profile of 100 RNA regulators, unpaired *t*-test was conducted to compare the gene expression between tumor and normal tissues. Notably, nearly 90% RNA regulators exhibited significantly higher expression in tumors than normal tissues (Figure 1A). Among the eight types of RNA modification regulators, all regulators in mcm^5^s^2^U, Ψ and A-to-I showed elevated expression in tumor tissues. The dysregulation of these RNA regulators may play key roles in the development of HCC.

The mutation profiles of 100 hundred regulators in HCC were depicted together. As a results, 94 of 371 samples (about 25.34%) showed RNA modification regulator mutations. M^5^C eraser TET1 and A-to-I writer ADARB2 had a mutation rate of 2%. About 40% RNA regulators had a mutation rate of 1% (Figure 1B). Genetic interaction analysis showed that mutation of m^5^C eraser TET3 was positively correlated with mutations of TRMT10B, ADAT2, PRRC2A, YTHDC1 and TET2. Mutation of m^6^A reader HNRNPA2B1 was positively correlated with mutations of WTAP, ELAVL1, MRM2, HNRNPC, HNRNPA1, YTHDC1, PRRC2A. Mutation of m^6^A writer ZC3H13 was positively correlated with mutations of TRMT10B, TET3, TRMT13, PRRC2A, YTHDC1, ADAR, YTHDC2 and ZCCHC4, while negatively correlated with mutations of METTL1 and CTU2 (Figure 1C).

To reveal the interactive relationships between proteins encoded by 100 RNA regulators, we constructed a PPI network using STRING and Cytoscape software. Correlations with adjusted *p*-value <0.05 and |r| ≥ 0.6 were shown in Figure 1D. We can see that these RNA regulators, especially regulators of m^6^A and m^5^C, showed close correlations with each other.

Results above collectively demonstrated that there were huge interaction and crosstalk between these 100 RNA regulators. They may form a complicated network to synergistically mediate the development and progression of HCC.

### Identification of different RNA modification patterns in HCC

**FIGURE 2:**
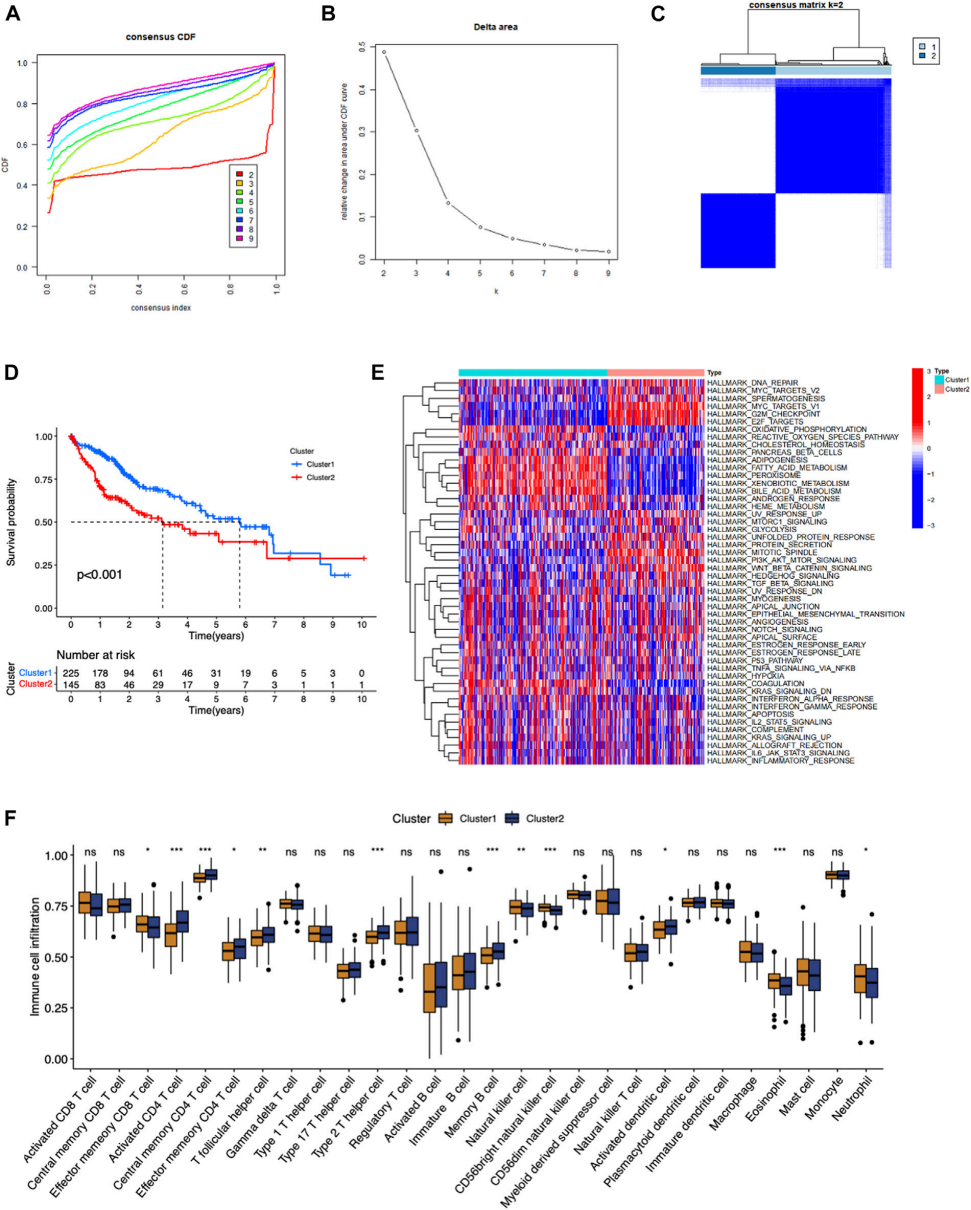
Identification of consensus clusters by eight types of RNA modification regulators in LIHC. **(A)** Cumulative distribution function (CDF) for *k* = 2–9. **(B)** The area under the CDF curve *k* = 2–9. **(C)** Consensus clustering matrix for *k* = 2. **(D)** Kaplan–Meier survival curve of two clusters. **(E)** The heatmap of the different cancer hallmarks enriched in two clusters. **(F)** The difference of the immune cells infiltrated in tumor microenvironments between two clusters.

In order to excavate the RNA modification patterns of HCC, consensus clustering analysis were conducted in 374 patients in TCGA-LIHC dataset. Patients in TCGA-LIHC dataset were separated into diverse clusters (*K* = 2–9) according to the expression of 100 RNA modification genes *via* an unsupervised consensus clustering analysis. The cumulative distribution function (CDF) curve and the area under the CDF curve was plotted, which indicated that the cohort was well distributed when *K* = 2 (Figures 2A–C). Kaplan-Meier survival analysis showed that patients in cluster 1 had a significantly better prognosis than patients in cluster 2 (*p* < 00.001) (Figure 2D). GSVA and ssGSEA enrichment analysis were performed to analyze biological process in two clusters. As shown in Figure 2E, most signaling pathways including DNA repair, MYC targets, G2M checkpoint, E2F targets were significantly enriched in Cluster 2. These may lead to the high degree of malignancy and poor prognosis of tumors in Cluster 2. Signaling pathways such as oxidative phosphorylation, reactive oxygen species pathway, cholesterol homeostasis and fatty acid metabolism were enriched in Cluster 1. There were more NK cells, eosinophils, neutrophils, effector memory CD8 T cells in Cluster 1, while more activated CD4 T cells, T follicular helper cells, type 2 T helper cell and effector memory CD4 T cells were accumulated in Cluster 2. This result implied two clusters had different immune infiltrating patterns.

Results above collectively suggested that 100 RNA modification regulators defined two RNA modification patterns in HCC, which exhibited different malignancy, immune microenvironment, and prognosis.

### Establishment and verification of RNA modification score in HCC

**FIGURE 3:**
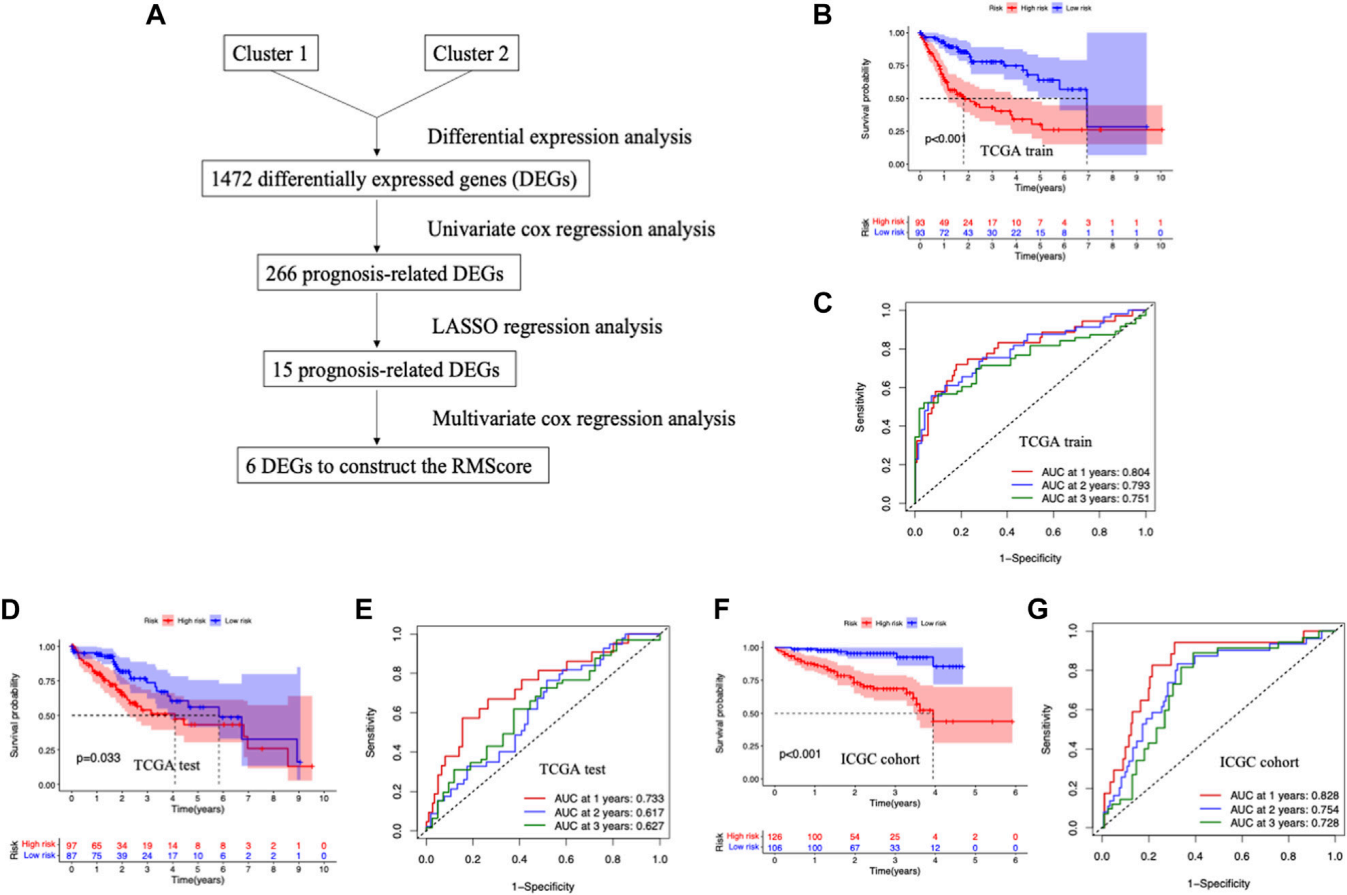
Development and validation of RNA modification related signature for HCC. **(A)** The workflow of the construction of the RMScore. **(B)** Survival curves stratified by the RMScore in the TCGA training cohort. **(C)** Receiver operating characteristic (ROC) curves of RMScore for predicting survival in the TGGA training cohort. **(D)** Survival curves stratified by the RMScore in the TCGA validation cohort. **(E)** ROC curves of RMScore for predicting survival in the TGGA validation cohort. **(F)** Survival curves stratified by the RMScore in the ICGC cohort. **(G)** ROC curves of RMScore for predicting survival in the ICGC cohort.

To further investigate the potential genetic alterations between two RNA modification patterns, differential expression analyses were carried out between two clusters (Figure 3A). A total of 1,472 differentially expressed genes (DEGs) were identified (Supplementary Table S2). Next, we divided the TCGA-LIHC queue into a training cohort and a verification cohort. Among the 1,427 DEGs, 266 genes were associated with the prognosis of HCC patients in training cohort by univariate COX regression analysis (Supplementary Table S3). Then, the LASSO regression analysis was conducted to further narrow the survival-related DEGs (Supplementary Table S4). Finally, six genes were identified by stepwise multivariate regression analysis and subsequently used to construct the RNA modification score (RMScore). The six genes identified were CYP2C9, CBX2, NDRG1, EPS8L3, FAM83D and MYCN. The RMScore = 0.1034*Exp of CBX2 + 0.0087*Exp of NDRG1 + 0.0392*Exp of EPS8L3 + 0.0517*Exp of FAM83D + 0.0596*Exp of MYCN−0.0031*Exp of CYP2C9.

By Kaplan-Meier analysis, we found that patients in low-risk group had a clear survival advantage over the high-risk group in training set (*p* < 0.001) (Figure 3B). The same survival advantage was also observed in the validation cohort (*p* = 0.033) and the ICGC cohort (*p* < 0.001) (Figures 3D, F). The time-dependent ROC curves were utilized in assessing the performance of RMScore, which showed the RMScore had good sensitivity and specificity in prognosis prediction (Figures 3C, E, G). These results suggested that the RMScore had good prediction ability for the survival of HCC patients.

### Independent prognostic value of RMScore

**FIGURE 4:**
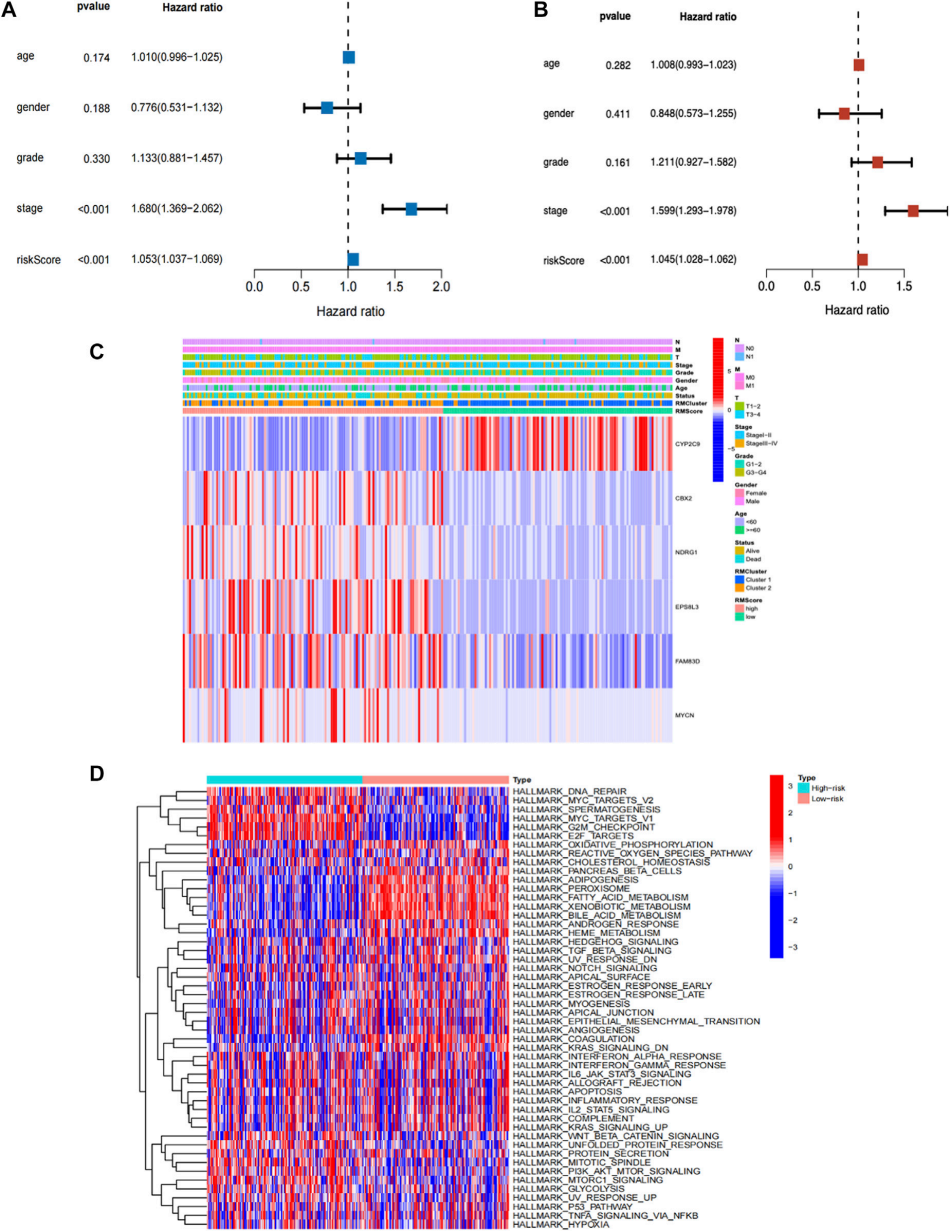
**(A)** Univariate Cox regression analysis and **(B)** multivariate Cox regression analysis verifying independent prognostic value of RMScore for the prognosis of HCC patients. **(C)** Heat map for the association of gene and clinicopathologic features with prognosis of HCC patients. **(D)** Visualization of biological processes analyzed by GSVA in two RMScore subgroups.

The RMScore together with clinicopathological factors, such as age, gender, grade, and stage were included for univariate and multivariate Cox regression analysis. In the univariate analysis, the RMScore was significantly related to the prognosis of HCC patients (hazard ratio [HR], 1.053; 95% confidence interval [CI], 1.037–1.069; *p* < 0.001) (Figure 4A). Multivariate analysis also demonstrated that the RMScore was an independent risk of the prognosis of HCC patients (hazard ratio [HR], 1.045; 95% confidence interval [CI], 1.028–1.062; *p* < 0.001) (Figure 4B). In the heatmap incorporating gene expression and clinicopathological traits, the majority of patients in Cluster 1 were gathered in low RMScore group, which demonstrated that the RMScore well represented the traits of patients in Cluster 1. Among the six gene which constructing the RMScore, only CYP2C9 was upregulated in the Cluster 1 as well as low RMScore group. The other five genes (CBX2, NDRG1, EPSBL3, FAM83D and MYCN) were upregulated in Cluster 2 as well as high RMScore group (Figure 4C). We explored the pathway enrichment associated with the RMScore to identify the internal mechanisms involved. Similar to the pathway enrichment results between Cluster 1 and Cluster 2, the high RMScore group was mainly enriched in signaling pathways related to DNA repair, MYC targets, G2M checkpoint, and E2F targets, while the low RMScore group was enriched in oxidative phosphorylation, reactive oxygen species pathway, cholesterol homeostasis and fatty acid metabolism (Figure 4D).

### Potential predictive biomarker for chemotherapy and target therapy

**FIGURE 5:**
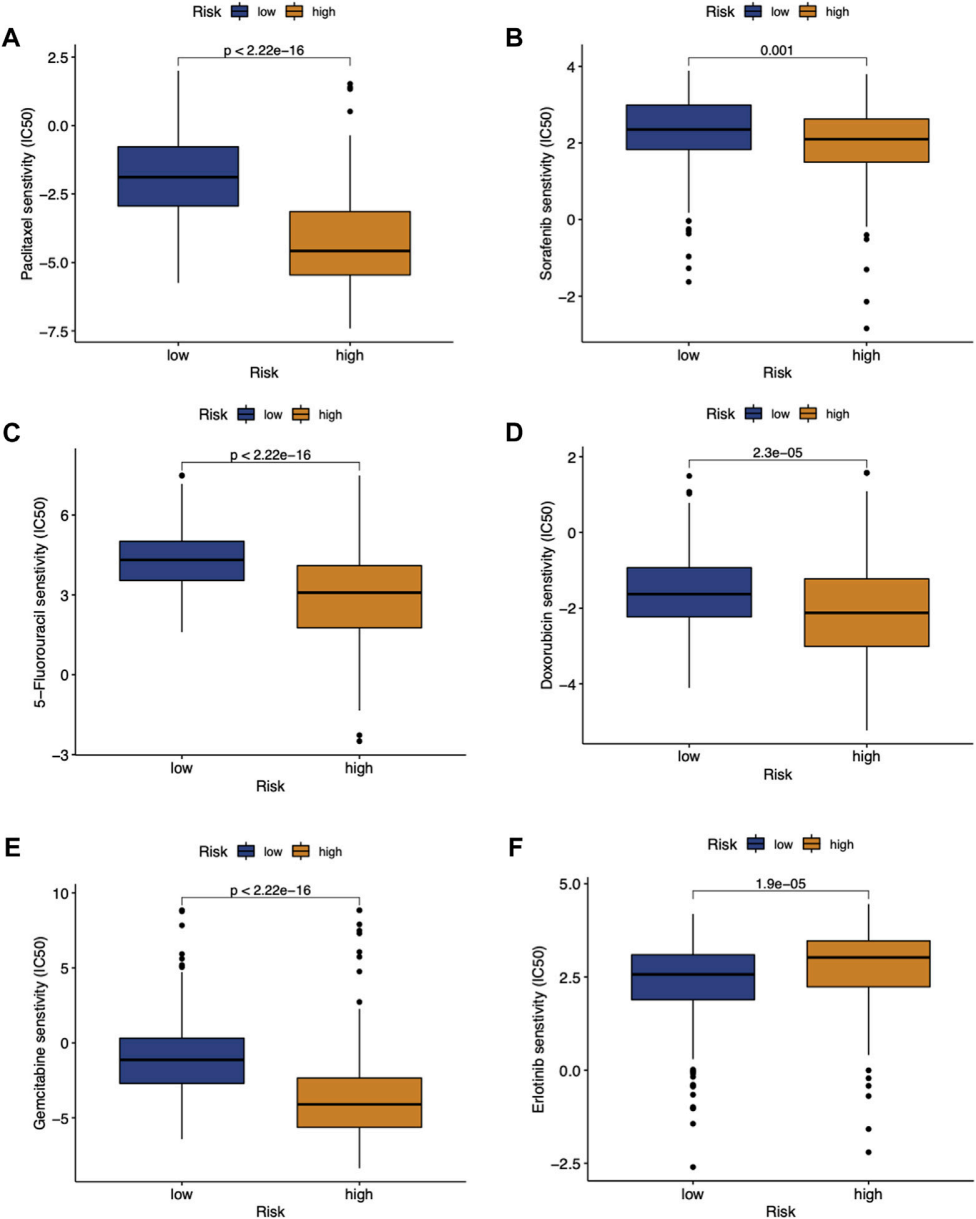
Estimated IC50 for paclitaxel **(A)**, sorafenib **(B)**, 5-flurouracil **(C)**, doxorubicin **(D)**, gemcitabine **(E)** and erlotinib **(F)** between high- and low-RMScore group.

We investigated the association between the RMScore and the sensitivity to chemotherapeutics in HCC patients. As a result, significant differences in the estimated IC50 between the two risk groups were observed for common chemotherapy or targeted drugs including paclitaxel (Figure 5A), sorafenib (Figure 5B), 5-flurouracil (Figure 5C), doxorubicin (Figure 5D), gemcitabine (Figure 5E) and erlotinib (Figure 5F). Lower RMScore was related to higher IC50 among paclitaxel, sorafenib, 5-flurouracil, gemcitabine, suggesting that patients with low RMScore were less sensitive to these treatments. However, patients with low RMScore had a lower IC50 to erlotinib, suggesting that they may be more sensitive to erlotinib than high RMScore patients.

### Development of a predictive nomogram

**FIGURE 6:**
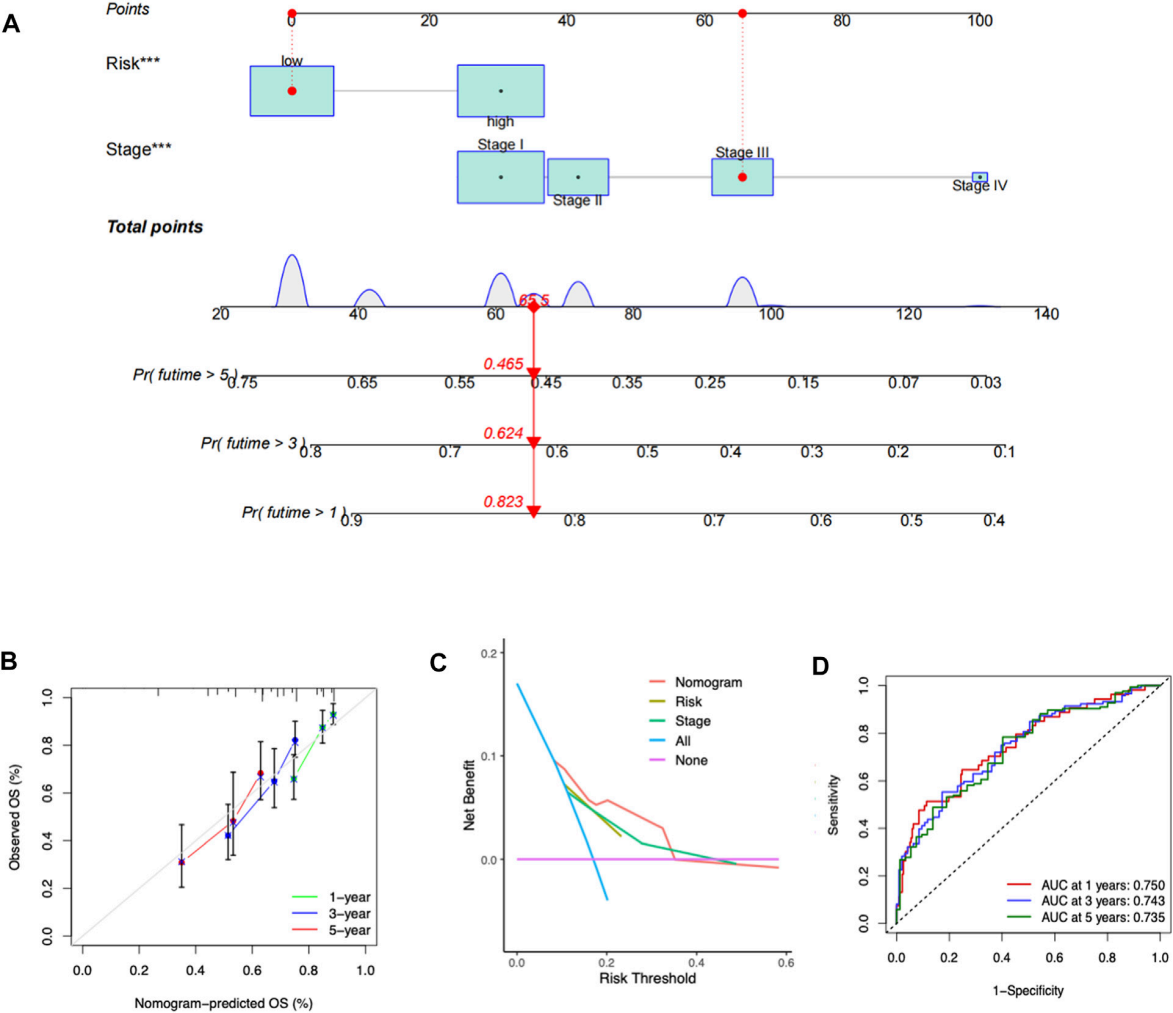
Construction of a nomogram by clinicopathological factors and RMScore. **(A)** Prognostic nomogram based on RMScore and tumor stage. **(B)** The 1-, 3-, and 5-year calibration plots of the nomogram. **(C)** DCA curves of the nomogram and other clinicopathological parameters. **(D)** The 1-, 3-, and 5-year ROC curves of the nomogram.

To facilitate the clinical applicability and availability of the RMScore, a predictive nomogram for 1-, 3-, 5-year OS combined with tumor stage and RMScore was developed (Figure 6A). The calibration curves showed that actual overall survival of patients was almost consistent with the predicted overall survival of patients (Figure 6B). The DCA plot suggested that the nomogram had a superior efficiency than RMScore in predicting patient survival outcomes (Figure 6C). The AUCs of the nomogram in the 1-, 3-, and 5-year ROC curves were 0.750, 0.743 and 0.735, respectively (Figure 6D).

## Discussion

The current interventions for HCC are not satisfactory, and more precise prognostic indicator and promising strategies need to be explored. Recent studies point out that RNA modifiers play vital roles in HCC cell proliferation, exacerbation, and metastasis (Zhu et al., 2021). However, these studies are mainly limited to several RNA modification regulators. The function of other kinds of RNA modification regulators and the interaction with each other remain to be elucidated.

Here, we systematically analyzed the expression, mutation, and the prognostic value of a total of one hundred RNA modifiers from eight types of RNA modifications in HCC. We found that most of the RNA modifiers were highly expressed in the tumor tissues compared to normal tissues. At the genetic level, nearly half of genes showed a least one kind of mutation. Most of the mutations were positively interacted with each other. These results suggested that eight types of RNA modifications might play important role in HCC.

Two RNA modification patterns with distinct prognosis were identified in HCC patients.

Then, we constructed RMScore to calculate the modification status of HCC patient. Patients with low RMScore showed better prognosis than patients with high RMScore. Similar to the pathway enrichment results in Cluster 1, pathway related to the cell cycle and proliferation, such as “DNA repair,” “G2M checkpoint,” “E2F targets” were all significantly enriched in the low RMScore group. All these results implied that the RMScore could well represent the modification status of the HCC patients.

There were some studies which documented different RNA modification patterns in various type of tumors, including HCC. For example, Qi et al. identified two distinct m6A modification patterns, one of which showed higher expression of m6A modification regulators and poor prognosis (Qi et al., 2020). Similarly, Li et al. reported two distinct clusters with different prognosis and clinical features based on the expression of 45 m6A/m5C/m1A regulated genes (Li et al., 2022). In addition, RNA modification patterns, especially m6A modification-related patterns, are also reported to have prognostic predictive value in lung cancer (Liu et al., 2020; Zhang et al., 2021), gastric cancer (Zhang et al., 2020), prostate cancer (Liu et al., 2021), kidney renal clear cell carcinoma (Li et al., 2021) and pancreatic adenocarcinoma (Wang et al., 2021b). In the current study, a total of one hundred cancer-related RNA regulators from eight-types of RNA modifications were included. We identified two subgroups with distinct cancer hallmarks, tumor microenvironments, and overall survival. These results showed the importance of other types of RNA modifications in HCC.

Finding optimal strategy to select patients most likely to benefit from available chemotherapeutic regimens can facilitate therapeutic decision-making. Recently, RNA modification was proved to be associated with sensitivity to anticancer therapies (Lan et al., 2021). For example, m^6^A writer METTL3 was documented to regulate sorafenib resistance though FOXO3-mediated autophagy in HCC (Lin et al., 2020). M7G writer WDR4 was demonstrated to play a role in sorafenib resistance by inducing CCNB1 translation in HCC. Herein, we used GDSC database to assess the differences in the sensitivity to various chemotherapeutics between the two RMScore groups. In our study, patients with high RMScore had a lower IC50, implying to be more sensitive to chemotherapy drugs including paclitaxel, sorafenib, 5-flurouracil, doxorubicin, mitomycin C and gemcitabine. The RMScore could be served as a good measure to predict the drug efficacy for clinicians.

Several limitations for our study should be recognized. Firstly, there were several cancer-related RNA modification regulators, such as m6Am regulators PCIF1 and METTL4, were not included in our analysis. This may lead to subtle deviations in our results, but does not affect the main conclusions. Secondly, due to the limited sample size, we still need more patients to verify the reliability of our conclusions. Thirdly, the potential mechanism of the role of genes contained in the RMScore was not based on experimental evidence, and required further *in vivo* and *in vitro* experiment. Last but not the least, as most of HCC patients were Caucasian (most were Hepatitis C virus-related HCC), whether the RMScore is also applicable to non-Caucasian is unknown. However, our study was the first study to give an insight into the mutation and expression of eight types of RNA modifications in HCC. This study paves the way for further investigations of the roles of eight types of RNA modifications in the HCC in the future.

## Data Availability

Publicly available datasets were analyzed in this study. This data can be found here: https://portal.gdc.cancer.gov; https://dcc.icgc.org.
